# Health-Related QoL of Hypertensive Patients in Bulgaria—Population-Based, Regional Pilot Study

**DOI:** 10.3390/medicina61081475

**Published:** 2025-08-17

**Authors:** Zornitsa Mitkova, Elena Dimitrova, Hristiana Tomova, Nikolay Gerasimov, Diyan Gospodinov, Konstantin Mitov, Stamen Pishev, Boryana Parashkevova, Guenka Petrova

**Affiliations:** 1 Department of Organization and Economy of Pharmacy, Faculty of Pharmacy, Medical University-Sofia, 1000 Sofia, Bulgaria; hristiana.tomova1@gmail.com (H.T.); kmitov@pharmfac.mu-sofia.bg (K.M.); gpetrova@pharmfac.mu-sofia.bg (G.P.); 2 National Cardiology Hospital Sofia, 1309 Sofia, Bulgaria; elena.sv@gmail.com; 3 Medical College, Trakia University, 6000 Stara Zagora, Bulgaria; nikolay.gerasimov@trakia-uni.bg; 4 Medical Center “Staykov” Burgas, 8000 Burgas, Bulgaria; diyan_gospodinov@abv.bg; 5 Diagnostic-Consulting Center I—Burgas, 8000 Burgas, Bulgaria; s_pishev@abv.bg; 6 Medical Faculty, Trakia University, 6000 Stara Zagora, Bulgaria; boryana.parashkevova@trakia-uni.bg

**Keywords:** hypertension, quality of life, EQ-5D-5L, Bulgarian patients

## Abstract

*Background and Objectives*: The goal of this study is to assess health-related quality of life (HRQoL) using the EuroQoL (EQ-5D-5L) and identify factors that might contribute to it. *Materials and Methods*: This is a real-life, observational study involving 234 hypertensive patients from Sofia and Burgas, conducted between January 2024 and July 2024. Patients were interviewed during their regular outpatient examinations and completed the questionnaire independently. *Results*: In total, 141 out of 234 respondents reported a visual analog scale (VAS) score of 70, and 152 reported a utility score of 0.7. The average VAS scores for males and females were 77.5 and 77.0, respectively, and the utility scores were 0.848 and 0.768, respectively. With advancing age, patients’ quality of life (QoL) decreased from 0.879 to 0.652 utility. Respondents with higher levels of education had higher utility scores (0.848 for bachelor’s/master’s degrees vs. 0.542 for primary education). With an increase in the number of concomitant diseases and prescribed medications, the QoL decreased (0.848 vs. 0.623 and 0.848 vs. 0.736, respectively). *Conclusions*: This first study of HRQoL using EQ-5D-5L among Bulgarian hypertensive patients revealed relatively high average VAS and utility scores. These results suggest that the disease is under control and that patients are being successfully treated and monitored. Factors such as comorbidity, residence, education, disability, and disease duration significantly affected and worsened patients’ HRQoL.

## 1. Introduction

Hypertension affects about 33% of adults aged 30–79 worldwide with an estimated prevalence of above 45% in 27 countries. The age-standardized prevalence increased slightly from 32% to 33% between 1990 and 2019. According to World Health Organization (WHO) data, 78% of adults with hypertension live in low- and middle-income countries [1].

Bulgaria as an East European country has high rates of smoking and metabolic syndrome both of which contribute to a high prevalence of cardiovascular diseases (CVDs) [2,3]. Approximately 75,617 deaths are attributed to CVDs [4]. The years of productive life lost due to hypertensive disability are about 9570 in Bulgaria [5]. Reimbursed expenditure for inpatient and outpatient treatment of CVDs in Bulgaria increased during 2017–2020, as did household expenditure for medications [6].

Improving disease control could positively impact long-term outcomes and reduce costs. However, data show that only about 42% of hypertensive adults are diagnosed and treated, and only one in five (21%) adequately control the disease [7]. Inadequate disease control could worsen the health-related quality of life (HRQol) of hypertensive patients and further compromise therapy [8].

HRQoL estimates how the patient’s health status could affect QoL and considers the utility associated with a specific health state. QoL is a broader term that includes more domains than the health ones, such as social interactions, spiritual domains, etc. [9].

One of the most frequently used tools for measuring HRQoL is the EuroQoL (EQ-5D) questionnaire, which has recently been expanded from EQ-5D-3L to EQ-5D-5L for greater sensitivity to mild changes in health [10,11]. EQ-5D-5L is a multidimensional concept that represents five QoL domains such as the patient’s physical, emotional, psychological, and social condition and provides five levels of possible answers according to severity of the condition. The EQ-5D-5L score can be converted into a utility index for use in cost-effectiveness analyses. This questionnaire allows the comparison of HRQoL across different diseases or populations, along with patient demographics such as age and gender [12]. Until now, it has not been applied to hypertensive patients in Bulgaria.

The widespread morbidity associated with hypertension in Bulgaria prompted our interest in assessing HRQoL using EQ-5D-5L and examining the factors that might influence it.

## 2. Materials and Methods

### 2.1. Study Design and Patient Recruitment

This is an observational, real-life study among patients with hypertension in Sofia and Burgas conducted from January 2024 to July 2024. Patients were interviewed during their regular examination in the outpatient setting and filled in the questionnaire independently.

The eligibility criteria included age over 18 years, clinically established hypertension as a primary diagnosis, and mental capability to respond. The exclusion criteria were age under 18, absence of clinically confirmed hypertension, and unwillingness to participate. The health-related quality of life (HRQoL) was assessed using the EQ-5D-5L questionnaire [13].

The translated version of EQ-5D-5L was provided by the Euro QoL group for Bulgaria. Each patient visiting the outpatient setting was invited to complete the informed consent form and the EQ-5D-5L questionnaire. Both sections of the EQ-5D-5L were used—the Visual Analogue Scale (VAS) and the EQ-5D-5L instrument. Demographic data collected included age, gender, level of education, place of residence, clinically confirmed disability, comorbidities, and polypharmacy (defined as the use of more than five medications), to explore their influence on HRQoL

### 2.2. Data Collection and Assessment

The collected data was inputted in Microsoft Excel, and a dataset for patient clinical and demographic characteristics was created with corresponding EQ-5D-5L answers. Descriptive statistics and the Kruskal–Wallis test were applied to assess correlations among the different patient groups and their characteristics.

### 2.3. Sample Size

The Raosoft calculator for sample size calculation is used [14]. The minimal number of patients required for a representative sample is 270 patients, whereas, in total, 234 participants were recruited in our study. Therefore, this study is considered a pilot survey for the population in both cities.

MedCalc ver.20.009 Software was used for statistical analysis.

### 2.4. Ethical Considerations

Ethical permission for the study was obtained from the Ethical committee of the Medical University of Sofia (decision No. 51/25 January 2024).

## 3. Results

### 3.1. Questionnaire Results

A total of 234 patients completed the questionnaire. The main demographic characteristics of the respondents are presented in Table 1.

Both gender groups are almost equally represented (Table 1). Nearly 95% of participants reported having a regular income either as employees or pensioners—an important factor in ensuring regular access to medication. Hypertension had been diagnosed within the past 10 years in the majority of respondents, and comorbidities were present in nearly all patients. Notably, approximately 60% of patients were on polypharmacy, which is consistent with the high prevalence of comorbid conditions.

The QoL of hypertensive patients, as measured through the VAS and EQ-5D-5L instruments, is presented in Table 2.

A VAS score above 60 was reported by 61% of respondents, while the remaining 39% scored below this threshold. Similarly, 75% of respondents reported a quality-of-life (QoL) score above 0.5.

**Figure 1:**
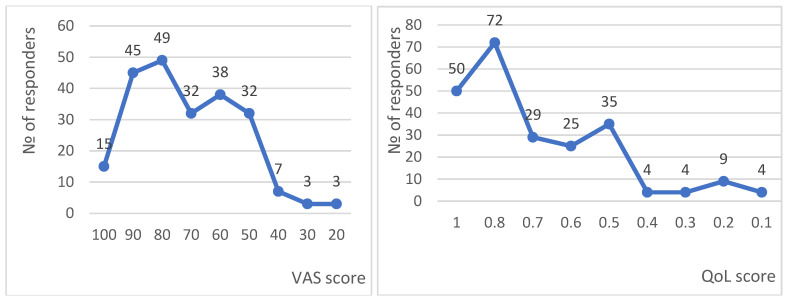
Distribution of responses per EQ-5D-5L scales.

The distribution of respondents according to VAS and EQ-5D-5L utility scores is presented in Figure 1. Notably, more patients rated their QoL higher using the EQ-5D-5L utility index than on the VAS scale. In total, 141 out of 234 respondents reported a VAS score of 70, and 152 reported a utility score of 0.7.

### 3.2. Statistical Analysis

Almost all demographic factors explored in the study impact the utility score in a statistically significant way (*p* < 0.05)—Table 3. Notably, while comorbidities significantly affected utility, polypharmacy (defined as the use of more than five medications) did not have a statistically significant impact (Table 3).

Utility scores declined with increasing age—from 0.879 in the group under 30 years to 0.652 in the group over 70 years. A corresponding increase in the median disability percentage was observed with age, which also negatively influenced utility.

Participants with higher education levels reported better utility scores (0.848 for bachelor’s/master’s degrees vs. 0.542 for primary education). Utility was higher among urban residents than in those living in rural areas (0.848 in cities vs. 0.592 in villages) and among those with a shorter duration of hypertension (0.863 for <10 years vs. 0.414 for >20 years).

With an increase in the number of concomitant diseases and number of prescribed medicines, utility decreased (0.848 vs. 0.623 and 0.848 vs. 0.736, respectively), but, as noted for the medicines, it was not statistically significant.

In terms of self-assessed VAS scores, only gender did not have a statistically significant impact (Table 4).

Logically, the VAS score was lower for male patients, the elderly above 70 years of age, pensioners, people with disabilities, low education, long-lasting disease with comorbidities, and those taking more than six medicines.

## 4. Discussion

In this study, we aimed to assess the health-related quality of life (HRQoL) of hypertensive patients in two major Bulgarian regions—Sofia and Burgas—using the EQ-5D-5L questionnaire. Out of Bulgaria’s 6.437 million residents, approximately 1.5 million live in Sofia and 0.389 million in Burgas, thus covering nearly one-third of the population. To the best of our knowledge, this is the first regional study to explore HRQoL in hypertensive patients using the EQ-5D-5L instrument in Bulgaria. The tool has been officially translated into Bulgarian, and we obtained permission for its use in scientific research [15].

Despite its advantages, few studies using EQ-5D-5L in the area of hypertension have been published. We found an average VAS score of 77.5 and 77.0 and a utility score of 0.848 and 0.768 for males and females, respectively. Those scores are relatively high, pointing out manageable hypertension. A Slovenian study reported an average EQ-5D-5L utility index of 0.7 and a VAS score of 70.9—both lower than those observed in our study [16].

Among Chinese hypertensive patients, the mean utility score was 0.85, with differences mainly based on gender, age, smoking status, duration of hypertension, and comorbidities [17]. In our study the utility index, especially for male patients was closer to the Chinese one. Mean VAS score and EQ-5D-5L indexes of hypertensive patients in Vietnam were estimated to be 71.48 and 0.94, respectively [18]. Lower values than the Bulgarian ones are found in Pakistan in hypertensive patients, with utility and VAS scores of 0.64 (±0.15) and 63.17 (±11.01), respectively, where the number of prescribed antihypertensive medicines had no effect on the patients’ quality of life, while patients with comorbidities reported lower EQ-VAS scores [19].

Patients who are aware of their hypertension tend to report lower scores in physical functioning and general health than those who are unaware of their condition [20]. However, appropriate treatment and awareness are crucial for maintaining mental and emotional well-being. While some studies found that hypertension does not significantly affect social, physical, or functional aspects, it often has a moderate impact on pain [21].

With advancing age, QoL and VAS declined significantly from 0.879 for 30-year-old individuals to 0.652 for 70-year-old individuals, and from 100 to 60 VAS score, respectively. A previous study in Bulgarian hypertensive patients using the EQ-5D-3L reported a slightly different perspective. It revealed that, overall, HRQoL is significantly lower among young hypertensive patients than in non-hypertensive adults of the same age, which is mainly due to often-experienced pain and anxiety. In the group of older patients (over 55), QoL is similar to that of non-hypertensive patients [22]. Our study findings confirm that disability affected the patients’ QoL (0.668 vs. 0.848) and VAS (60.0 vs. 80.0) compared with the non-disabled patients.

A study in Ethiopia found that HRQoL among hypertensive patients declined with age, duration of treatment, low social support, physical inactivity, presence of comorbidities, and marital status [23]. In our study, we similarly found that age, gender, comorbidities, place of residence, education level, disability, and years since diagnosis significantly influenced HRQoL in Bulgarian hypertensive patients. A longer duration of illness was associated with a lower QoL (0.863 in patients diagnosed less than 10 years ago vs. 0.586 in those diagnosed more than 20 years ago), which could be related to factors such as medication side effects or treatment fatigue [24,25].

Non-pharmacological interventions in hypertensive patients, especially those focused on lifestyle changes have been shown to improve quality of life, particularly in the physical domain. Adherence to antihypertensive therapy positively affects both physical and mental well-being and is essential for achieving better clinical outcomes. A previous study revealed very high adherence rates among Bulgarian hypertensive patients, which may partly explain the relatively high QoL values found in our study [26]. Along with hypertension, other conditions may also affect the participants’ QoL, as well as their lifestyle habits. The following comorbidities were considered as important and assessed using the participants’ demographic characteristics: diabetes—74 patients (31%), other cardiovascular disease—111 patients (47%), chronic lung disease—15 patients (6.4%), patients reporting any other disease—108 (46.1%). Further studies are needed to evaluate their individual contributions.

The main limitations of this study are its regional scope, the relatively small sample size, and the absence of a national hypertension registry in Bulgaria, which limits the generalizability of the findings. Nevertheless, our statistical analysis supports the reliability of the real-world data obtained. Larger, nationally representative studies are needed to validate these conclusions. Further studies are also needed to evaluate the QoL of chronic diseases such as myocardial infarction, diabetes, and obesity. The other limitation of our study is that it does not evaluate the QoL at the moment of the presence of high blood pressure. We evaluated hypertensive patients in outpatient settings despite the reason for their visit to physicians. This approach was due to the fact that the methodology of QoL measurement requires patients to answer the questionnaires by themselves and not in the presence of healthcare specialists.

## 5. Conclusions

This first EQ-5D-5L-based study of HRQoL in Bulgarian hypertensive patients revealed relatively high average VAS and utility scores. These findings suggest that hypertension in this patient population is generally well- controlled and that patients are receiving effective treatment and follow-up. However, factors such as comorbidity, place of residence, education level, disability, and duration of illness were found to significantly and negatively affect the patients’ HRQoL.

## Figures and Tables

**Table 1:** Demographics and clinical characteristics of enrolled patients.

Parameter	№. of Patients (%)
Gender	
Male	115 (49.15)
Female	119 (51.85)
Disability	
Yes	44 (18.8)
No	190 (81.2)
Employment status	
Employed	118 (50.42)
Pensioner	103 (44.02)
Not employed	13 (5.56)
Years with hypertension diagnosis	
<10	104 (44.45)
Between 10 and 20	74 (31.62)
>20	56 (23.93)
Years with comorbidities	
<10	91 (38.89)
Between 10 and 20	83 (35.47)
>20	60 (25.64)
Number of diagnosed comorbidities	
0	75 (32.05)
1	91 (38.89)
≥2	68 (29.06)
Number of prescribed medicinal products	
≤2 medicinal products	88 (37.61)
3–5 medicinal products	112 (47.86)
>5 medicinal products	34 (14.53)

**Table 2:** VAS and utility score of the QoL reported by the patients.

VAS Score	% of Patients	Utility Score	% of Patients
100	6.70	1	21.37
90	20.09	0.8	30.77
80	21.88	0.7	12.39
70	14.29	0.6	10.68
60	16.96	0.5	14.96
50	14.29	0.4	1.71
40	3.13	0.3	1.71
30	1.34	-0.3	0.85
20	1.34	0.2	3.85
-	-	0.1	1.71

**Table 3:** Statistical analysis of different factors impacts on utility score.

Parameter	Patient Groups	Descriptive Statistics, Median	Kruskal–Wallis’s Test, Significance Level (*p*)
Gender	Female (n = 115)	0.848	*p* = 0.003439
	Male (n =119)	0.768	
Age	<30 years age	0.879	*p*< 0.000001
	30–50 years age	0.864	
	50–70 years age	0.808	
	>70 years age	0.652	
Occupation	Unemployed	0.843	*p*< 0.000001
	Pensioner	0.679	
	Employed	0.877	
Disability	Yes	0.668	*p* = 0.000033
	No	0.848	
Education	Primary	0.542	*p* = 0.000004
	Secondary	0.795	
	Master or bachelor	0.848	
Residence	Village	0.592	*p* = 0.000076
	City	0.848	
Duration of diagnosis (years)	<10	0.863	*p* < 0.000001
	10–20 years	0.837	
	>20 years	0.586	
Number of concomitant diseases	None	0.848	*p* = 0.000047
	1 disease	0.848	
	2–4 diseases	0.656	
	>4 diseases	0.623	
Number of medicines	≤2 medicines	0.848	*p* = 0.222548
	3–5 medicines	0.774	
	>5 medicines	0.736	

**Table 4:** Statistical analysis of different factors’ impact on VAS score.

Parameter	Patient Groups	Descriptive Statistics, Median	Kruskal–Wallis’s Test, Significance Level (*p*)
Gender	Female (n = 115)	77.5	*p* = 0.259248
	Male (n =119)	70.0	
Age	<30 years age	100.0	*p*< 0.000001
	30–50 years age	85.0	
	50–70 years age	75.0	
	>70 years age	60.0	
Occupation	Unemployed	75.0	*p*< 0.000001
	Pensioner	60.0	
	Employed	80.0	
Disability	Yes	60.0	*p* = 0.000001
	No	80.0	
Education	Primary	52.5	*p* = 0.000104
	Secondary	75.0	
	Master or bachelor	80.0	
Residence	Village	55.0	*p*< 0.000001
	City	80.0	
Duration of diagnosis	<10	80.0	*p*< 0.000001
	10–20 years	70.0	
	>20 years	55.0	
Number of concomitant diseases	None	80.0	
	1 disease	72.5	*p*< 0.000001
	2–4 diseases	60.0	
	>4 diseases	47.5	
Number of medicines	≤2 medicines	80.0	*p* = 0.000012
	3–5 medicines	70.0	
	>5 medicines	60.0	

## Data Availability

The data that support the findings of this study are available upon reasonable request from the corresponding author (Z.M.).
